# Patient safety and patient assessment in pre-hospital care: a study protocol

**DOI:** 10.1186/s13049-016-0206-7

**Published:** 2016-02-12

**Authors:** Magnus Andersson Hagiwara, Lena Nilsson, Anneli Strömsöe, Christer Axelsson, Anna Kängström, Johan Herlitz

**Affiliations:** Centre for Prehospital Research, Faculty of Caring Science, Work Life and Social Welfare, University of Borås, SE-501 90 Borås, Sweden; Department of Anaesthesiology and Intensive Care, Linköping University, SE-581 85 Linköping, Sweden; Department of Medical and Health Sciences, Linköping University, SE-581 85 Linköping, Sweden; School of Health, Care and Social Welfare, Mälardalens högskola, Box 883, SE-721 23 Västerås, Sweden

**Keywords:** Adverse events, Patient assessment, Pre-hospital care, Patient safety

## Abstract

**Background:**

Patient safety issues in pre-hospital care are poorly investigated. The aim of the planned study is to survey patient safety problems in pre-hospital care in Sweden.

**Methods/Design:**

The study is a retro-perspective structured medical record review based on the use of 11 screening criteria. Two instruments for structured medical record review are used: a trigger tool instrument designed for pre-hospital care and a newly development instrument designed to compare the pre-hospital assessment with the final hospital assessment. Three different ambulance organisations are participating in the study. Every month, one rater in each organisation randomly collects 30 medical records for review. With guidance from the review instrument, he/she independently reviews the record. Every month, the review team meet for a discussion of problematic reviews. The results will be analysed with descriptive statistics and logistic regression.

**Discussion:**

The findings will make an important contribution to knowledge about patient safety issues in pre-hospital care.

## Background

When the *Committee on Quality of Health Care in America*, *Institute of Medicine* published the report entitled *To Err Is Human: Building a Safer Health System* [1], the issue of patient safety was highlighted in health-care organisations around the globe. The report revealed that somewhere between 44,000 to 98,000 people died in American hospitals because of preventable harmful incidents. A later study [2] has estimated that the actual figure is up to 400,000 deaths each year in American hospitals. These are alarming reports and highlight patient risks in acute care hospitals. Patient safety is a problem also in an international perspective. There are estimates that 10 % of patients admitted to a hospital are exposed to harmful incidents [3]. Swedish hospitals have patient safety problems that correspond to the international figures. A structured medical record review from 2003–2004 [4] revealed that 12.3 % of the reviewed records contained adverse events and 70 % of the adverse events were regarded as preventable. Another report based on a structured medical record review from 2012, including hospitals all over Sweden, performed by the Swedish Association of Local Authorities and Regions [5], found an adverse event rate of 14 %.

To our knowledge, there are no reports of patient safety issues in pre-hospital care in Sweden and there is also a shortage of investigations of pre-hospital care from a global perspective [6]. In a pre-hospital patient safety project in Canada [7, 8], clinical reasoning and decision-making were identified as the most important issues in connection with pre-hospital patient safety. There are other studies which support this view [9–11]. None of these studies are based on structured medical record reviews. The rationale for clinical reasoning and decision-making as the main threat to patient safety is the rapid development of pre-hospital care and the fact that pre-hospital providers do not have the appropriate education or the tools for the complex pre-hospital work [9]. Examples of the more complex pre-hospital assessments and interventions are advanced cardio-pulmonary resuscitation (CPR), as well as the early identification and transport bypass protocols for patients with myocardial infarction and stroke [7]. The development of pre-hospital care in Sweden is similar [12] and the question is whether the system has followed developments in areas like education, guidelines, protocols, equipment and accreditations. It is therefore important to investigate prehospital provider’s clinical reasoning and decision-making so appropriate system changes can be made. Since 2005, all ambulances in Sweden have been required to be run by at least one registered nurse. There is no requirement of special training in prehospital care and the number of nurses and requirement for special training differ between different organisations. The ambulances can be staffed either with one nurse and one emergency medical technician or with two nurses [13]. Another identified patient safety issue is compliance with guidelines in pre-hospital care. Several studies have shown that compliance with pre-hospital guidelines is sometimes low [14–18]. The reasons for low compliance have not been fully investigated, but there are studies showing that poorly designed guidelines and protocols could have an effect on compliance [19, 20].

The threats to patient safety in pre-hospital care are probably not comparable to patient safety issues in hospital and the problem is underestimated [21]. In order to acquire more knowledge of patient safety issues in pre-hospital care, there is a need for research on the frequency and the type of adverse events, as well as in areas such as safety cultures, error reporting, clinical reasoning, decision-making and education [22, 23].

## Methods/design

### Theoretical framework

The study is inspired by Reason’s theories of human error [24]. Reason divides errors in organisations into active and passive failures. Active failures are the errors made by the front-end people in an organisation. In health-care organisations, they include errors made, for example, by nurses, doctors and paramedics in their contact with the patient. Active failures can often be explained by underlying latent failures. Latent failures occur when the organisation has failed to build a system containing defence layers to prevent active failures from taking place [25]. There are many examples of latent failures in a health-care organisation; they include poorly adapted equipment, a lack of resources, insufficient safety culture, poorly adapted cognitive support and poor working schedules [26]. The goal of our study is to describe the frequency and types of adverse event in pre-hospital care to help pre-hospital organisations create better defence layers to prevent active failures committed by the exposed front-end providers. In order to prevent adverse events, it is important to build a safer system and not to put any blame on those working in a deficient safety organisation.

We use patient safety terminology described in a framework formulated by Runciman et al. [27]. Patient safety is described as *the reduction of risk of unnecessary harm associated with healthcare to an acceptable minimum*; error is the *failure to carry out a planned action as intended* and is divided in errors of commission, described as using an inadequate plan but the implementation proceeds as planned, and omission, which is described as situations in which the plan is good but failures occur in the execution of the plan. Violation is the term for *deliberate deviation from an operating procedure, standard or rule* [27].

### Study aims

The overall study aim is to survey patient safety problems in pre-hospital care in Sweden.

The specific aims are:

1. To describe the frequency and types of adverse events in pre-hospital care
2. To investigate whether the level and type of adverse events in pre-hospital care differ between men and women, different age groups, different groups of patients, or types of ambulance mission
3. To investigate whether the level and type of adverse events in pre-hospital care differ between the included organisations and if there are relationships to level of education and experience among the prehospital providers
4. To investigate the agreement between the pre-hospital assessment of the patient with the final diagnosis in hospital

### Study design and setting

The study is a structured medical record review based on the use of 11 screening criteria. The study has been implemented in three pre-hospital organisations in Sweden. Two organisations are located in the Västra Götaland Region and one organisation in the County of Dalarna. The rationale for the sampled organisations is that they include both urban and more rural districts. One organisation represents an urban district, one a mix of urban and rural districts and one organisation represents a rural district where the majority of the patients is living outside a city.

### Study material and sampling

The study material comprises pre-hospital and hospital medical records. Each month, the pre-hospital and hospital medical records, connected to 30 ambulance missions in each included organisation, which fulfil the inclusion criteria, are collected. Institute for Healthcare Improvement recommends a sample of 20 to 40 records each month [28]. To ensure random collection, a random number generator is used. If there is any excluded record among the 30 randomly collected records, the procedure is repeated until 30 records which fulfil the inclusion criteria and lack exclusion criteria are found. This procedure is repeated until the study has lasted for 12 months with data collection starting on 1 September 2015 and ending on 31 August 2016. In all, the study will include medical record reviews of 1,080 ambulance missions.

### Inclusion criterion

The inclusion criterion is ambulance missions which include any kind of patient assessment and care.

### Exclusion criteria

The exclusion criteria are:

1. Patient < 18 years
2. Ambulance missions without patient contact
3. Ambulance missions which provide secondary support to another ambulance
4. Ambulance missions which are labelled as transportation between health-care facilities

The rationale of excluding children in this investigation is that we believe that patient safety issues in this patient category are unique [29] and there is a need for an investigation with special emphasis on pre-hospital paediatric patient safety. The reason to exclude secondary support to another ambulance is that those records often are non-informative since the ambulance crew who have the main responsibility are in charge for the documentation. When it comes to excluding transportation between health-care facilities, we believe that patient safety issues in those cases are unique and there is an ongoing project with special focus on those missions.

### Instrument

The study is using two different instruments in the structured medical record review. The first instrument is a newly developed trigger tool designed for helicopter emergency care developed by Patterson et al. [30]. In a short pilot study, we found that the instrument was also suitable for ground-based ambulances. To date, we have not found any published studies that have used this instrument. The instrument consists of 11 triggers (Table 1). The rater screens the ambulance medical record to identify whether one or more triggers can be found in the record. If any trigger can be identified, the next step is to categorise the trigger in five different categories and, in the last step, the seriousness of the incident is classified as no adverse event, possible adverse event and identified adverse event.

**Table 1:** Description of triggers and categories in the trigger tool. For full instrument see Patterson et al. (28)

Triggers (11)
Documentation triggers
1. Missing, incomplete, or unclear documentation for the following: chief complaint, physical assessment, vital signs, haemodynamic monitoring, allergies, pertinent history or medications, patient condition at handoff.
Operational & patient movement triggers
2. Time from initial patient contact to transfer of care exceeds accepted standards.
3. Injury to patient or team member during patient encounter/transport.
4. Request for additional resources, personnel, or supervisor due to change in patient condition.
Patient condition triggers
5. A worsening trend in patient haemodynamic or mental status indicators.
6. Cardiac arrest during transport.
Intervention & medication triggers
7. Use of any of the following interventions during patient care: cardioversion, defibrillation, transcutaneous pacing, advanced airway attempt, surgical airway, intraosseous (IO), chest decompression, chest tube.
8. Failure of any intervention or procedure during patient care.
9. Use of following medications or fluids: blood products, vasopressors, inotrope, naloxone.
10. Evidence of deviation from standard of care by performing an intervention or administering a medication that appears to be outside protocol or failure to perform an intervention or provide a medication that is within the standard of care.
11. Medication error.
Categories (5)
1. Actions By Patient: The adverse event was the result of action(s) by the patient.
2. Actions By Provider: The adverse event was the result of action(s) or inaction(s) by the crew.
3. Medical or Vehicle Equipment: Failure of the equipment, failure to troubleshoot and correct common problems with the equipment, or failure to remove defective equipment from service.
4. Environmental/Scene Factors: Factors that may result from weather conditions or factors on the ground/scene (or other). This includes temperature, light and scene safety.
5. Undetermined by Chart Review: The proximal cause of the adverse events (regardless of severity) cannot be determined by the information available in the chart.
Classification (3)
1. No adverse events
2. Adverse event present – potential for harm
3. Adverse event – harm identified

The other instrument has been developed for use in the present study. The instrument is designed to compare the pre-hospital assessment with the final hospital assessment. The assessment should be categorised in one of five major categories according to the final diagnosis, depending on a) the seriousness of the disease (life threatening or not); b) the precision of the final diagnosis (reflecting a disease such as myocardial infarction or a symptom such as chest pain or a more diffuse condition (such as poor general condition) and c) the availability of the final assessment (sometimes not available).

Thus, the five major categories according to the final diagnosis were: A) a definite life threatening diagnosis (i.e. myocardial infarction) ; B) a definite non-life threatening diagnosis (i.e. herpes zoster) ;C) the final diagnosis is expressed as a symptom (i.e. chest pain) ; D ) the final diagnosis is expressed in non-specified terms (i.e. deteriorated general condition) and E) information on the final assessment is not available. This means that the first category includes life threatening conditions in contrast to the following four categories.

The next step is to evaluate the association between the pre-hospital assessment and the final assessment. For A and B there are six possibilities. 1) The field diagnosis is in agreement with the final diagnosis. 2) The field diagnosis is not in agreement with the final diagnosis. 3) The field assessment is a typical symptom when related to the final diagnosis; for example, chest discomfort, if the final diagnosis is a myocardial infarction. 4) The field assessment is an atypical symptom in relation to the final diagnosis; for example, dyspnoea, if the final diagnosis is myocardial infarction. 5) The field assessment is a more unusual symptom in relation to the final diagnosis; for example, abdominal pain, if the final diagnosis is myocardial infarction. 6) The field assessment describes the patient’s problem in a less specific way; for example, “problems with the circulation” or “problems with the airways.”

For C and D there are only five subcategories since the final assessment is not specified in a diagnosis (Table 2). These subcategories are:

**Table 2:** Instrument to compare the pre-hospital assessment with the final hospital assessment

A	A defined final diagnosis classified as life threatening. Example: myocardial infarction
1)	The field diagnosis is in agreement with the final diagnosis. Example: suspected myocardial infarction or unstable angina pectoris
2)	The field diagnosis is not in agreement with the final diagnosis. Example: pneumonia
3)	Typical symptoms related to the final diagnosis are described. Example: chest discomfort
4)	Atypical symptoms related to the final diagnosis are described. Example: dyspnoea
5)	More unusual symptoms related to the final diagnosis are described. Example: abdominal pain
6)	Field assessment as a non-specified organ system. Example: circulation
B	A defined final diagnosis classified as not life threatening. Example: bronchitis
1)	The field diagnosis is in agreement with the final diagnosis. Example: pneumonia
2)	The field diagnosis is not in agreement with the final diagnosis. Example: congestive heart failure
3)	Typical symptoms related to the final diagnosis are described. Example: fever and coughing
4)	Atypical symptoms related to the final diagnosis are described. Example: dyspnoea
5)	More unusual symptoms related to the final diagnosis are described. Example: vertigo
6)	Field assessment as a non-specified organ system. Example: airways
C	The final diagnosis is expressed as a symptom. Example: dyspnoea
1)	The field diagnosis is in agreement with the final symptom. Example: congestive heart failure
2)	The field diagnosis is not in agreement with the symptom. Example: stroke
3)	Field symptoms and final symptoms are in agreement. Example: breathing problem
4)	Field symptoms and final symptoms are not in agreement Example: chest discomfort
5)	Field assessment as a non-specified organ system. Example: circulation
D	The final diagnosis is expressed as a non-specific assessment. Example: psychiatric insufficiencies
1)	The field diagnosis is in agreement with the symptom. Example: psychosis
2)	The field diagnosis is not in agreement with the symptom. Example: stroke
3)	Field symptoms are in agreement with final assessment. Example: anxiety
4)	Field symptoms and final assessment are not in agreement. Example: chest discomfort
5)	Field assessment presented as a non-specified organ system. Example: nervous conditions
E	The patient is transported to a level of care other than the hospital and no final diagnosis is available.
1)	Patient refereed to primary care
2)	Patient stayed at home or at a nursing home with extended home care
3)	Patient stayed on scene with self-care advice

1) The field assessment is a diagnosis in agreement with the final assessment. 2) The field assessment is a diagnosis not in agreement with the final assessment. 3) The field assessment is a symptom in agreement with the final assessment. 4) The field assessment is a symptom not in agreement with the final assessment. 5) The field assessment describes the patient’s problem in a less specific way as described in A and B subcategory 6.

**Fig. 1:**
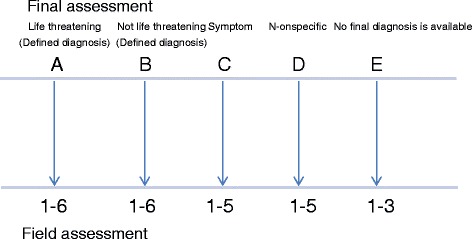
Evaluation of the relation between prehospital field assessment and hospital final assessment

Category E includes patients in whom there is no information on the final assessment since these patients were not taken to a hospital. Three subcategories are involved describing various modes of care including 1) primary care 2) extended home care and 3) self-care on scene (Table 2, Fig. 1).

Finally, we will not only relate the final assessment to the field assessment in terms of diagnosis or symptoms on scene. We will also relate the final assessment to the assessment of risk on scene. Risk on scene will be assessed according to the Rapid Emergency Triage and Treatment (RETTS) model [31].

The instrument is recently developed and not validated. There are problems when trying to compare prehospital assessments with the final assessment in the hospital. One reason is the amount of differential diagnoses related to one single initial symptom. For example; the symptom “chest discomfort” relates to a number of various differential diagnoses. Another difficulty is that the prehospital health care providers sometimes describe the patient in terms of symptoms and sometimes in terms of diagnoses. It is therefore often problematic to judge the clinical consequence of the prehospital assessment. When the final assessment includes several diagnoses, one that could be linked to the EMS mission will be chosen. If none of the final diagnoses can be linked to the EMS mission, the patient will not be included in the analysis.

Finally the term life threatening final diagnosis will include any of the following diagnoses: myocardial infarction, unstable angina pectoris, TIA/stroke, unconsciousness, septicaemia, aortic rupture, aortic dissection, any form of shock, pulmonary embolism, heart failure including pulmonary oedema, failing heart conducting system, cardiac arrest and intoxication.

The aim of the present study is to test and validate the instrument so that it can be used in future studies with the intention to evaluate prehospital assessments.

### Personnel

In every included ambulance organisation, one rater has the main responsibility for data collections and medical record reviews. All the raters are experienced ambulance nurses with specialist education in prehospital care and at least 10 years’ experience from the prehospital field. They have all participated in two sessions of rater training. The rest of the research team comprises two registered nurses, a researcher in pre-hospital care and two medical doctors with a research background in pre-hospital care and patient safety. The raters conduct medical record reviews each month. Records they find difficult to rate are saved for a monthly review meeting which all the participants in the team attend. The meetings are live or a mix of live and video meetings. At these meetings, the team discuss difficult medical records and make a final decision based on consensus. In order to assess inter-rater reliability, 10 % of the medical records are reviewed by two raters.

### Statistics

Inter-rater reliability between two raters will be calculated with kappa coefficients (к) [32]. To determine frequencies and types of adverse event in the reviewed medical records, descriptive statistics will be used and, to detect any possible differences between different patient categories, odds ratios will be calculated with logistic regression. All statistical analyses will be performed using the SPSS 21.0 statistical software program (SPSS Inc, Chicago, IL).

### Ethics

The main ethical problem in the present study is threats to personal integrity. The problem will be approached by careful data handling so that people who are not involved in the study will not be able to see any of the content in the collected medical records. The collected medical records will not be stored in a computer database but only in the form of paper copies which are locked in a special room at the university. When collecting the ambulance medical records, the number of the ambulance or the name or number of the ambulance personnel will be invisible. The study has been approved by the Regional Ethics Committee, Gothenburg, Sweden (Dnr: 047–15).

## Discussion

The present study has several important aspects. The first aspect is that there is a lack of studies that survey the patient safety problems in pre-hospital care [8]. It is probably not possible to generalise the frequency and types of adverse event in hospital to the pre-hospital context. What makes pre-hospital care so unique is that the care is provided far away from medical support. The care is given in a changing and sometimes difficult environment. The pre-hospital health-care providers assess patients with many different symptoms and conditions which sometimes have a high degree of acuity. The pre-hospital health-care providers have different levels of education and experience [19]. In addition, pre-hospital health-care providers use different kinds of vehicle and many types of equipment not relevant to the hospital setting. Other issues which make pre-hospital emergency care so unique is that the care is provided in an unstable environment, due to an uncontrolled volume of patients, a variable level of acuity, a lack of information, time sensitivity, stress and fatigue [33]. According to Reason [26], the human factor is responsible for up to 90 % of accidents in hazardous technologies and it is reasonable to suppose that this is true even in pre-hospital care.

In error management, there are two dominant approaches. The first approach is the person approach that focuses on the active failures. This approach sees the cause of the error in the individual. Errors happen because people are reckless, lazy and careless or have poor morale. Typical strategies to reduce error in this approach are poster campaigns, new procedures and threats of disciplinary measures [25]. This approach is a part of the blame-and-shame culture and is unfortunately still a common approach in health-care organisations [34]. The second approach is the system approach. The main theme in the system approach is that humans are imperfect and errors are natural when humans are involved in a task. If errors are expected, it is the system which should be built so that errors can be avoided or the consequences of the errors can be minimised [25]. This approach is common in high-reliability organisations, such as the aircraft industry, shipping, military organisations and nuclear power plants. To be able to build defence layers in a system to prevent errors, it is important to survey the frequency and the types of error in the organisation.

The second aspect is that knowledge of adverse events in pre-hospital care can be used to develop interventions (defence layers) with the goal of preventing adverse events. Examples of these interventions can include educational interventions, decision support, the development of better equipment, the development of better working schedules, the development of feedback systems and working to improve patient safety culture.

The third important aspect of the present study is the opportunity to test methods and instruments that can be used for continuous quality improvement measurements in pre-hospital organisations and to measure the effect of various interventions. Being able to measure processes and evaluate changes is important in quality improvement work [35].

### Limitations and requirements

Every type of study involving reviews of medical records has limitations. There is a risk that the medical record does not reflect the reality in an objective manner [36]. The reason is that data in medical records are several steps from the patient. For example, in pre-hospital care, the electronic medical records in many organisations are completed after the hand-over in the emergency department and sometimes a long time after the event, introducing the risk of recall bias. A study has shown that most electronic pre-hospital records are unavailable when emergency physicians make crucial medical decisions [37]. The use of well-developed instruments for structured reviews of medical records is a way to minimise bias [38].

When using reviews of medical records as a research method, a number of requirements should be met: a) the method that is being used should answer the research question; b) all medical records that could be included should have an equal probability of selection; c) the variables to be collected should be determined in advance and documented in a coding manual; d) the data collection should be systematic; e) the reviewers should be well trained for the task; f) there should be organised meetings at which the reviewers’ conflicts can be resolved and coding rules reviewed and g) IRR measurements should be made [36].
